# Advantage of Clinics

**Published:** 1871-04

**Authors:** Geo. F. Mills

**Affiliations:** Brooklyn, N. Y.


### ADVANTAGE OF CLINICS.

BY GEO. F. MILLS, BROOKLYN, N. Y.

It is surprising and gratifying as well, to observe the avidity with which clinics are received wherever properly introduced. No dentist who loves advancement but feels a growing desire as he attends clinical gatherings to see them more numerously established throughout the whole field of the profession.

There is something done in this direction within the walls of the dental colleges, but these only reach a small portion of the profession at large, for it is well known that there are hundreds of practicing dentists who as yet know little if anything of the happy influences of professional association. To reach these is the great desideratum, and hence arises the question, why the anxiety for those without? I answer, we are our brother's keeper. It is incumbent upon all who are in the light to bring others into it.

We do not get so near the truth, which is but the light, in our discussions alone as we can in our contact with actual demonstration. There the principle and the method of application are coupled, and no one will deny the practical benefit.

One noticeable result of clinical gatherings is the respect they almost invariably create in those who attend them, for each other's opinions; and criticisms are more pleasantly made. Men do not always speak with their tongues what they know in their hearts, and we want, therefore, teachings that will take hold upon the conscience. If they do not they are powerless for good.

The dentist that attends clinics will begin to apply the measure to his own standard of knowledge, and soon discover his deficiencies. Fraternal feeling is developed by them, and they impress facts on the mind of one who has just come from seclusion.

No doubt but clinics are held in private offices that have not come to the general knowledge for the reason that some earnest but modest spirit is fearful that he may be called forward. But every city should have its public clinic, where all may receive the benefit. Clinics should be held in the country towns once a month; or better, once a week. Some may urge that the country towns can not effect this as readily or as well as cities. "Where there is a will there is a way."

Some men will not associate because they have not been introduced. I once violated the code by introducing myself to a dentist, and I was punished in a few months afterwards by him sending me patients who paid me over six hundred dollars for services rendered.

I believe that immense good would result from the movement that has been suggested, namely, to send out competent men to go where dentists could be gathered together, and there teach by word and by demonstration, and I believe it possible for the right men on such a mission to create such an enthusiasm that the cry would come from all quarters, "come over and help us."

